# Co-Application of Organic and Ca, Mg, Zn Fertilizers Reshapes Depth-Stratified Arbuscular Mycorrhizal Fungal Communities in Orchard Soil

**DOI:** 10.3390/jof12080543

**Published:** 2026-07-23

**Authors:** Hong Li, Xin Jiao, Youshan Wang, Na Sun

**Affiliations:** 1 Institute of Plant Nutrition, Resources and Environment, Beijing Academy of Agriculture and Forestry Sciences, Beijing 100097, China; wangyoushan5150@163.com; 2 College of Resources and Environmental Sciences, China Agriculture University, Beijing 100193, China; jiaoxin@cau.edu.cn

**Keywords:** soil depth stratification, canopy status, full soil profile, subsoil microbiome, depth-dependent AMF

## Abstract

Arbuscular mycorrhizal fungi (AMF) are crucial symbiotic microorganisms in terrestrial ecosystems, playing a vital role in maintaining orchard soil health and productivity. However, how organic–and Ca, Mg, Zn fertilizers co-application affect vertical stratification and ecological functions of arbuscular mycorrhizal fungi (AMF) in perennial fruit orchards remains unclear. Based on a five-year in situ peach trial, we established three fertilization regimes: low- (LWF), medium- (MWF), and high-input (HWF) regimes. We systematically analyzed the AMF community structure, diversity, and their correlations with soil physicochemical properties, as well as peach tree physiology, fruit yield, and quality across two soil depths: 0–20 cm (topsoil) and 20–40 cm (subsoil). HWF significantly inhibited AMF root colonization rates and spore density (*p* < 0.05), while reducing community α-diversity AMF α-diversity (*p* < 0.05), characterized by the enrichment of genera such as *Glomus* and a decrease in the relative abundance of *Rhizoglomus*. Redundancy analysis (RDA) identified available Zn (AZn) and Mg (WMg) as key drivers of this restructuring. Integrating RDA results into depth-specific partial least squares structural equation models (PLS-SEM), we found that subsoil AZn/WMg indirectly boosted yield by reshaping AMF composition (β = 0.34, *p* = 0.006), mediated via improved canopy status (NDVI, PRI). Total effect analysis confirmed the dominant role of subsoil pathways. These findings challenge the prevailing topsoil-centric view of soil microbial ecology and underscore the importance of considering the full soil profile when evaluating the impacts of agricultural practices on beneficial symbionts. We conclude that sustainable management strategies should account for depth-dependent AMF responses to maintain both productivity and belowground biodiversity across the entire rooting zone.

## 1. Introduction

AMF can form obligate symbioses with the roots of most plants [1,2], delivering critical ecosystem services such as enhanced phosphorus and nitrogen acquisition, improved water uptake, and increased tolerance to drought, salinity, and pathogens [3,4,5]. In return, plants allocate 4–20% of photosynthetically fixed carbon to them [6]. This mutualism underpins soil aggregation, nutrient cycling, and overall agroecosystem resilience [7,8]. However, conventional agricultural intensification—including high mineral fertilizer inputs, tillage, and monoculture—has eroded arbuscular mycorrhizal fungi (AMF) diversity and suppressed colonization intensity, potentially undermining the long-term sustainability of soil functions [8,9]. In practice, organic fertilizers were widely used to enhance soil functions, yet their impacts on AMF remain inconsistent: some studies reported stimulation [10,11,12], while others showed neutral or even inhibitory outcomes [13,14]. This variability suggests that the type, composition, and management context organic fertilizers critically modulate AMF responses—but the mechanisms underlying these effects remain unclear. This uncertainty is particularly pronounced in perennial orchard systems. Unlike annual crop ecosystems, orchards feature deep-rooted trees and are subject to distinct, often intense, management regimes [15,16].

In Chinese peach (*Prunus persica* (L.) *Batsch*) production, for instance, fertilization strategies increasingly emphasize the combined application of medium and Ca, Mg, Zn nutreints to prevent physiological disorders such as leaf yellowing and poor fruit set, which are common in high-phosphorus or sandy soils [17,18,19]. While AMF are known to facilitate the acquisition of micronutrients like Zn, Cu, and Fe [20,21,22,23], it remains unknown whether the direct application of these medium and micronutrients alters the “demand-side” of the symbiosis. Specifically, does direct nutrient supplementation reduce the host plant’s reliance on the mycorrhizal pathway, thereby shifting the AMF community structure. The interactive effects of organic fertilization and targeted medium/micronutrient supplementation on AMF communities remain largely unexplored [24].

Furthermore, current understanding of AMF responses to management is biased toward topsoil layers. AMF communities are vertically stratified, with distinct taxa adapted to the specific edaphic conditions and root niches found at depth [12,16]. The subsoil (>20 cm) acts as a critical reservoir for water and nutrients and potentially harbors more stable, stress-tolerant microbial communities [25,26]. In deep-rooted perennial systems like peach orchards, where roots can extend beyond 60 cm [27], subsoil AMF may be essential for sustaining nutrient supply during critical phenological stages. However, traditional fertilization is typically concentrated in the topsoil layer, and most studies focus on short-term topsoil responses [10,12,13,14]. Ignoring the deep soil profile may lead to a “hidden” loss of functional diversity and a misunderstanding of how orchard management impacts the total soil microbiome [8,10,28,29].

To address these gaps, we examined AMF root colonization, spore density, community composition at two depths (0–20 cm and 20–40 cm), as well as productivity attributes (foliar spectral indices, fruit yield, and quality) in a 14-year peach (*P. persica*) orchard under a 5-year field experiment: low-input conventional fertilization (LWF), moderate organic fertilization (MWF), and organic plus medium and micronutrient fertilization (HWF). We tested the following hypotheses: Organic fertilization may suppress AMF symbiosis, and co-application of organic and Ca, Mg, Zn fertilizers will exacerbate the suppression; Fertilizations may affect AMF abundance and diversity, especially functional genera; Fertilization effects on AMF will be depth-dependent due to altered soil nutrient availability; Shifts in AMF functional genera (e.g., *Rhizoglomus*, *Glomus*) driven by fertilization regimes will influence intricate trade-offs between fruit yield and quality.

## 2. Materials and Methods

### 2.1. Study Site and Experimental Design

**Figure 1:**
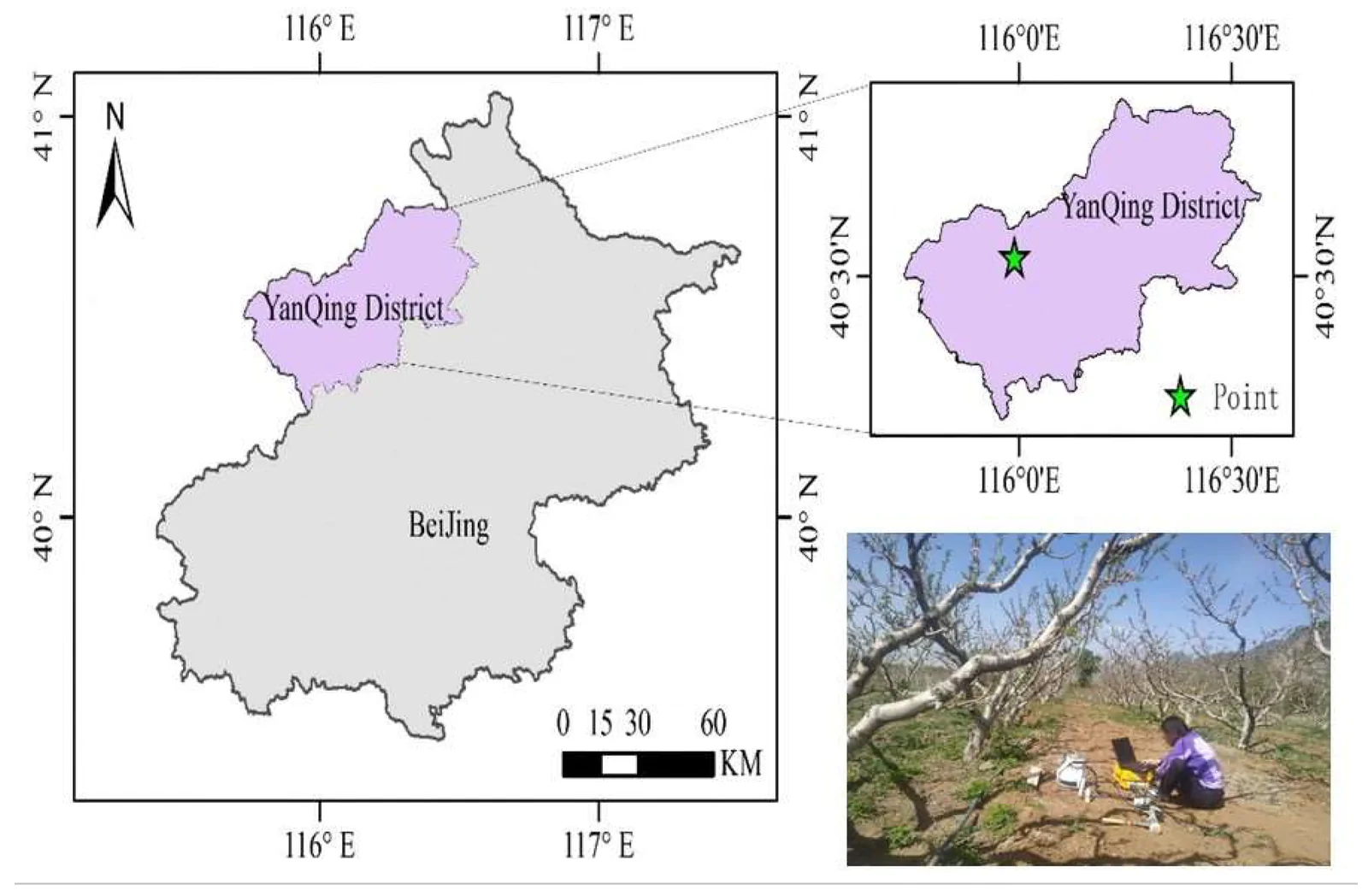
The location of our field experimental peach orchard.

The study was conducted in a 20-ha peach orchard founded in 2008 on meadow cinnamon soil (Typic Hapli-Udic Argosols) located in the Yanqing District of Beijing, China (115.99° E, 40.53° N) (Figure 1). This region is characterized by a warm temperate continental monsoon climate, with an annual mean temperature of 8.5 °C and an average annual precipitation of 443 mm. The experimental site features uniform topography, ensuring consistent environmental conditions across the area. The orchard has been cultivated with the early-ripening peach variety ‘Chunxue’ since 2010, with a planting density of 4 m between plants and 3 m between rows. Soil characteristics before the treatment phase in 2016 are detailed in Table S1.

At the end of October each year since 2016, a randomized block design was used to implement three fertilization regimes: LWF, MWF, and HWF (Table 1):

In the field experiment, a trench, 1.2 m in length, 0.6 m in width, and 0.6 m in depth, was excavated on the western side of each selected peach tree (Figure S1a). The excavated soil was stratified into two depth intervals—0–20 cm and 20–40 cm, and then completely mixed with the assigned fertilizer regime before being backfilled into the trench. Sixty-three physiologically uniform peach trees, whose varieties and tree ages were the same, were selected and randomly assigned to three experimental treatments (Figure S1b). Throughout the experimental period, pest management and routine orchard maintenance practices were conducted in accordance with local standard procedures.

### 2.2. Soil and Roots Sampling

Soil and plant samples were collected on 19 July 2021, during the key fruit maturation stage. For each replicate plot, rhizosphere soil samples were collected from the 0–20 cm and 20–40 cm layers at a distance of 1.5 m west of the central tree trunk (the sample point location is determined by the radius of the tree crown projection). Each sample was a composite of three soil cores. We collected 42 samples (3 treatments × 7 duplicates × 2 depths). A portion of the fresh soil was immediately stored at −80 °C for molecular analysis, another portion was stored at 4 °C for the assessment of spore density, and the remainder was air-dried for physicochemical analysis. At the same time, fine roots (diameter < 2 mm) were sampled from the representative peach tree by carefully excavating the root system with a shovel. Before excavation around each tree, the shovel was thoroughly disinfected with 97% ethanol-soaked wipes to prevent cross-contamination. Five to six intact fine root segments were selected per tree, immediately placed in an insulated icebox, and transported to the laboratory for subsequent analysis.

### 2.3. Soil Physicochemical Properties

Conventional techniques were utilized for analyzing the soil. Soil bulk density (SBD) and porosity (SP) were assessed using the core technique [30]. Soil water content (SWC) was determined gravimetrically [31], while soil clay content (SFC) was measured using the pipette method [32]. Soil pH was measured in a 1:2.5 (*w*/*v*) suspension of soil and water. Soil organic carbon (SOC) was measured using the potassium dichromate wet oxidation technique [33]. Available nitrogen (AN) was evaluated through the alkaline-hydrolysis diffusion technique [34]. Extracted with 0.5 M NaHCO_3_ (pH 8.5), available phosphorus (AP) was quantified colorimetrically [35]. Extractable potassium (AK) was obtained using 1 M NH_4_OAc (pH 7.0) and measured through flame photometry [35]. Water-soluble calcium (WCa) and magnesium (WMg) were extracted using 1 M CH_3_COONH_4_ and analyzed via ICP-OES [36]. Available zinc (AZn) was obtained using DTPA and measured through ICP-MS [37]. Every analysis was conducted on seven separate replicates.

### 2.4. Foliar Spectral Traits and Fruit Quality Attributes

During the peach maturity stage, foliar spectral reflectance was measured between 10:00 and 11:00 a.m. on clear, sunny days using a portable spectrometer (PolyPen RP-410, Photon Systems Instruments, Drasov, Czech Republic). The following indices were calculated from the reflectance data: Normalized Difference Vegetation Index (NDVI), Greenness Index (GI), Optimized Soil-Adjusted Vegetation Index (OSAVI), Photochemical Reflectance Index (PRI), Normalized Pigment Chlorophyll Index (NPCI), Modified Chlorophyll Absorption Ratio Index (MCARI), and Structure Insensitive Pigment Index (SIPI). Equations for the calculation of these indices are listed in Supplementary Table S2. For each replicate, one representative peach tree was selected. From each of the four cardinal directions (east, west, south, and north) of the canopy, three healthy, sun-exposed branches were randomly chosen. On each selected branch, the 7th or 8th fully expanded functional leaf from the apex was identified, and spectral reflectance was recorded on three such leaves per direction. The mean reflectance of these leaves was calculated to represent the spectral signature of that canopy direction. Subsequently, a canopy-level spectral reflectance value for the entire tree was derived by computing a weighted average based on the number of branches per cardinal direction.

On 19 July 2021, fruit samples were collected for the attribute assessment. For each replicate, six fully mature and representative peach fruits were harvested from each of three canopy orientations—south, north, and either east or west—of a selected representative tree, resulting in a total of 18 fruits per tree. Before sampling, uniform fruit thinning had been conducted across all experimental trees to ensure a consistent fruit load per tree. Under this standardized management regime, individual fruit weight was considered a reliable proxy for yield estimation at the tree level. Fruit quality analysis included single fruit weight (SFW, weighing method), transverse and vertical diameters (TD, VD, ruler method), total soluble solids (TSS, measured by digital refractometer), soluble sugars (SS, determined by the anthrone-sulfuric acid method), titratable acidity (TA, by titration with 0.1 M NaOH), vitamin C (VC, via 2, 6-dichlorophenolindophenol titration), and nitrate content (NC, by salicylic acid nitration method).

### 2.5. AMF Colonizaton Characteristics and Spore Density

Fine root samples of each duplicate obtained from 2.2 were rinsed, cut into 0.5–1.0 cm segments, and processed for AMF visualization: cleared in 20% KOH, acidified with 5% acetic acid, stained with 5% ink in acetic acid, and destained in water [38]. Thirty root segments were randomly selected from each sample and placed on a slide. We counted the AMF infection characteristics under a 100× optical microscope using the cross-line method. Colonization parameters—F (root mycorrhizal colonization rate), M (root mycorrhizal colonization density), m (density of mycorrhizal colonization in colonized root segments)—were quantified using the Trouvelot method via Mycocalc (http://GGG2.dijon.inra.fr/mychintec/Mycocalc-prg/MYCOCALC.EXE, accessed on 30 April 2026) [39].

Spores and sporocarps were extracted from 20 g dry soil using wet sieving and sucrose flotation centrifugation (500 g L^−1^) [40], collected in gridded Petri dishes, and counted under a dissecting microscope (Olympus SZXILLK200, Olympus Corporation, Tokyo, Japan). Sporocarps were counted as single spores; results are reported as spore density (SD).

### 2.6. DNA Extraction, Amplicon Sequencing, and Bioinformatics

DNA was extracted from 0.5 g soil and the ITS gene amplicon sequences were amplified using the primers AML1F (ATCAACTTTCGATGGTAGGATAGA) and AML2R (GAACCCAAACACTTTGGTTTCC) [41]. The second PCR was conducted using primers AMV4.5NF (AAGCTCGTAGTTGAATTTCG) and AMDGR (CCCAACTATCCCTATTAATCAT) [42]. These primer pairs were selected in terms of reproducibility and precision to describe the AMF communities [43]. Amplicon sequencing was conducted using the Illumina MiSeq platform (Illumina, San Diego, CA, USA) at Majorbio Bio-Pharm Technology Co., Ltd., Shanghai, China. The raw sequencing data have been deposited in the NCBI Sequence Read Archive (SRA) under the BioProject accession number PRJNA835607.

Bioinformatic processing was conducted on the Majorbio Cloud Platform (www.majorbio.com). It included primer/barcode trimming, quality filtering (Q > 20), and clustering into OTUs at 97% similarity (UPARSE). OTUs were classified against the Silva database (RDP classifier, ≥70% confidence). *Glomeromycota* OTUs were further refined against the MaarjAM database (≥97% similarity, ≥95% alignment length, e-value < 1 × 10^−50^); unmatched sequences were BLAST (https://blast.ncbi.nlm.nih.gov/, accessed on 30 April 2026) against INSD (≥90% similarity, ≥90% alignment length). Non-AMF sequences were removed. OTUs with <5 reads were excluded, yielding 80 high-quality AMF OTUs.

### 2.7. Statistical Analyses

Before analysis, normality and homogeneity of variance tests were performed on each group of data using the Shapiro-Wilk method. Difference in Canopy Physiological Performance and Productivity Traits were analyzed using one-way ANOVA with Tukey’s HSD post hoc test in IBM SPSS Statistics (v25). Alpha-diversity, including diversity (Shannon and Simpson indices) and richness (Chao1 and ACE indices), was evaluated using Mothur (v1.31.2). Differences in alpha-diversity measures and soil properties across treatments and soil depths were assessed via two-way ANOVA with Tukey’s HSD post hoc test in IBM SPSS Statistics (v25). Beta-diversity at the genus level was computed based on Bray–Curtis dissimilarity and visualized through principal coordinates analysis (PCoA) in R (v3.3.1), followed by the PERMANOVA method for differential significance testing. LEfSe analysis implemented via the R microeco package (v17.0) was employed to identify differentially abundant taxa within the arbuscular mycorrhizal fungal (AMF) community and to evaluate the effect of fertilization using linear discriminant analysis (LDA) scores. This facilitated the detection of significant AMF biomarkers across fertilization regimes and soil depths. Prior to analysis, all variables were standardized by converting to Z-scores. A redundancy analysis (RDA) was performed for relationships between the AMF community and fruit attributes in CANOCO 5.0. The manual forward-selection procedure was used to determine the significance of soil characteristic variables (*p* < 0.05) using a Monte Carlo test with 499 permutations. All figures were generated in Origin 2021.

## 3. Results

### 3.1. Organic and Ca, Mg, Zn Fertilizers Enhance Canopy Physiological Performance and Productivity Traits

Canopy spectral indices revealed that fertilization significantly influenced the physiological status of peach (*P. persica*) trees (Table 2). In comparison to LWF, MWF significantly increased the NDVI (*p* < 0.05) and GI (*p* < 0.05), and lowered MCARI (*p* < 0.05), indicating greater canopy greenness and chlorophyll content. However, HWF triggered a more notable physiological response: each of the spectral indices assessed—NDVI, GI, OSAVI, PRI, and NPCI—was significantly higher compared to LWF (*p* < 0.05). Notably, HWF enhanced NPCI by 59.26% and increased PRI by 40%, reflecting improved photosynthetic efficiency and pigment control under HWF.

The enhancements at the canopy level seemed to improve fruit morphology and nutritional quality (Table 3). Both MWF and HWF significantly increased fruit TD and VD compared to LWF (*p* < 0.05). Although MWF had no significant impact on SFW, HWF considerably raised it to 196.04 g (*p* < 0.05). Concurrently, HWF achieved the lowest NC (90.4 mg·kg^−1^), a 58% higher VC content, and a 35.7% reduction in titratable acidity relative to LWF. SS and TSS were also maximized under HWF.

### 3.2. Fertilization Affects Arbuscular Mycorrhizal Symbiosis

**Figure 2:**
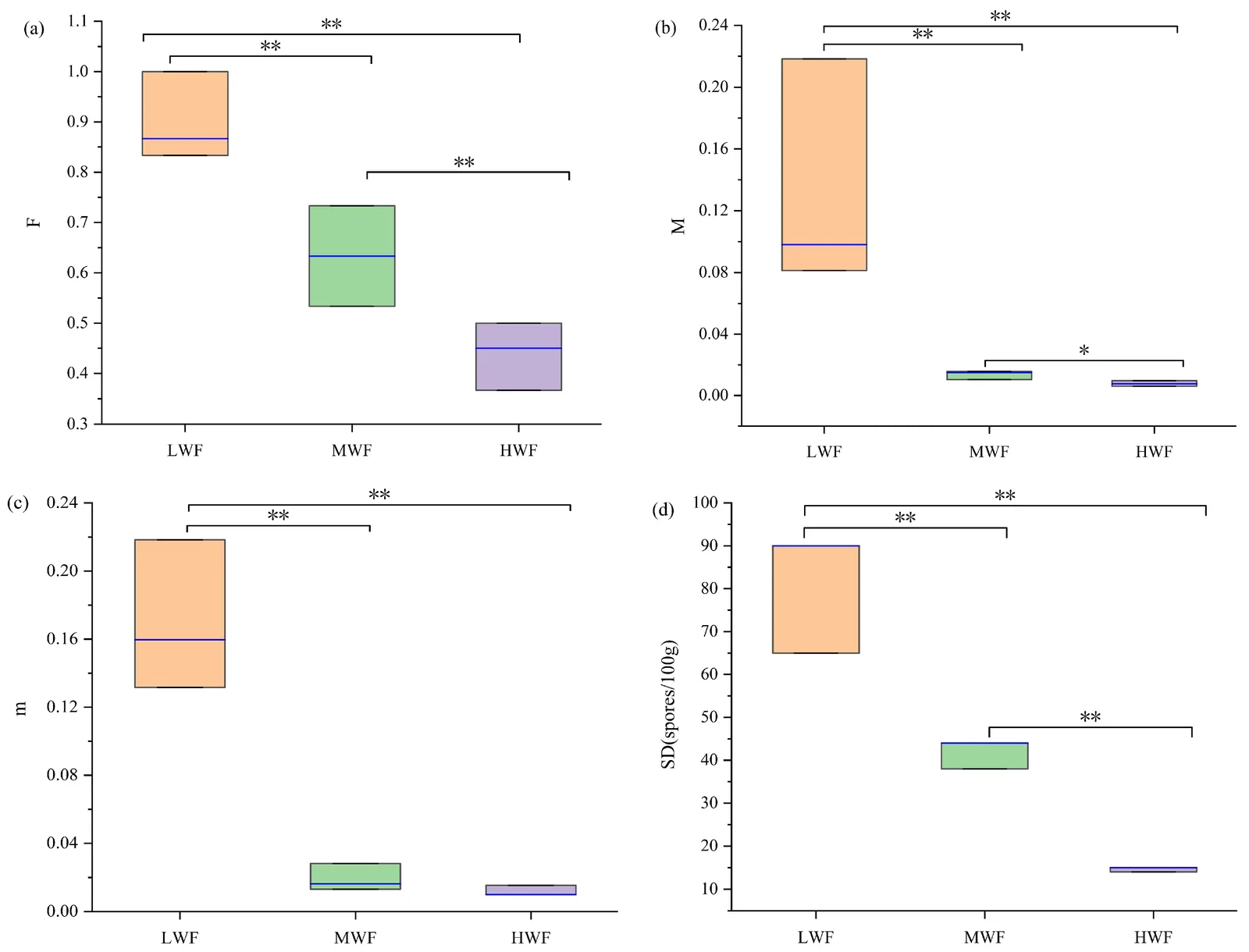
AMF root colonization characteristics and soil SD under different fertilization regimes (panel (**a**) = F, panel (**b**) = M, panel (**c**) = m, panel (**d**) = SD). Data are presented as mean ± SE of seven replicates. Significant effects among regimes are indicated with asterisks: *, *p* < 0.05; **, *p* < 0.01 (one-way ANOVA with Tukey’s HSD test). The coefficient of variation (CV) values of F, M, m and SD were 6.96%, 10.23%, 9.64%, and 21.76%, respectively.

Despite the aboveground benefits, intensified fertilization markedly disrupted the symbiotic relationship between peach roots and AMF. Root colonization frequency (F %) and intensity (M %) were significantly reduced under both MWF and HWF relative to LWF (Figure 2). Specifically, MWF decreased F by 28.6% and M by 24.3% (*p* < 0.01), whereas HWF induced a more severe suppression—F declined by 51.2% and M by 98.2% (*p* < 0.001). Similarly, SD of AMF in rhizosphere soil was reduced by 37.5% under MWF and 70.3% under HWF (*p* < 0.01). Given that SD reflects the propagule bank and reproductive potential of AMF, these findings suggest that organic, and Ca, Mg, Zn fertilization may impair long-term mycorrhizal resilience and recovery potential of AMF communities.

### 3.3. Fertilization Drives Depth-Stratified Shifts in AMF Alpha- and Beta-Diversity

Alpha-diversity analyses revealed that nutrient enrichment differentially affected AMF communities across soil depths (Figures S2 and S3). In both topsoil (0–20 cm) and subsoil (20–40 cm), the HWF treatment significantly reduced OTU richness (Chao1, ACE) and Shannon diversity while increasing Simpson dominance (*p* < 0.05), indicative of community homogenization under high nutrient availability.

**Figure 3:**
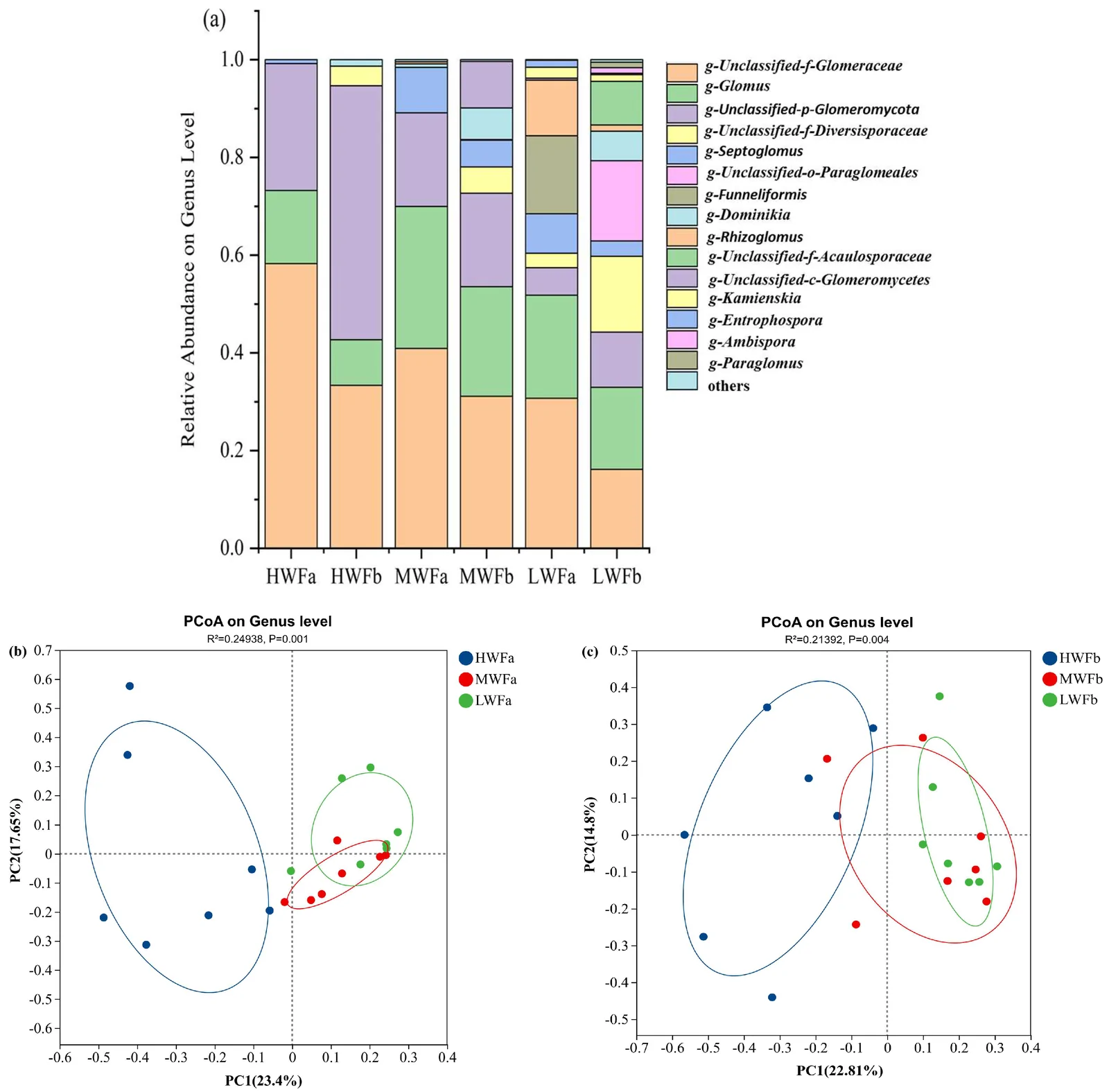
Relative abundance of AMF taxa in different soil layers of peach tree rhizosphere. Figure (**a**) shows the taxonomic composition of AMF communities in the soil. Genera with an abundance below 0.01 are combined into ‘Others’. LWFa is the control treatment; MWF is the organic fertilizer treatment; HWF is the treatment with combined application of organic fertilizer and and Ca, Mg, Zn fertilizers. Subscript a represents the topsoil, and b represents the subsoil. It is the same as the figure below. Figures (**b**,**c**) show the principal coordinate analysis (PCoA) of AMF genus-level community composition, where (**b**) is for the topsoil and (**c**) is for the subsoil.

Our results revealed AMF community composition exhibited strong vertical stratification (Figure 3a). Across both soil layers, the AMF community was dominated by *unclassified* (*aff. Glomeraceae*, *Glomerales*), *Glomus*, and *unclassified* (*aff. Glomerales*, *Glomeromycetes*, *Glomeromycota*), collectively accounting for over 50% of relative abundance, even 74.8% in HWF. Fertilization intensity drove significant shifts in AMF taxonomic composition. Compared to LWFa, HWFa and MWFa significantly increased the relative abundance of *Glomus* and significantly reduced the relative abundance of *Rhizoglomus* and *Kamienskia* (*p* < 0.05), but there was no significant difference between HWF and MWF (Figure S4a). Compared to LWFb, HWFb, and MWFb significantly reduced the relative abundances of *Rhizoglomus* and *Kamienskia* (*p* < 0.01), and MWFb significantly increased the relative abundance of *Dominikia* (Figure S4b). Principal Coordinates Analysis (PCoA) confirmed significant treatment-driven divergence in AMF assemblages in both layers (PERMANOVA: topsoil R^2^ = 0.249, *p* = 0.001; subsoil R^2^ = 0.214, *p* = 0.002), with HWF causing the greatest compositional shift along primary axes (Figure 3b,c). The sample clusters of three regimes showed different aggregations; samples under HWF were significantly more distant from LWF than those under MWF in the ordination space. This pattern was consistent across both soil layers, indicating that fertilization exerts a stronger influence on AMF community structure than soil depth alone.

LEfSe analysis identified depth- and treatment-specific indicator taxa (Figure S5). In the topsoil, *Dominikia* and *Entrophosporaceae*—taxa associated with high organic carbon—were enriched under organic inputs. Conversely, the subsoil was characterized by genera such as *Rhizoglomus* and members of the order *Glomerales* that were related to stress tolerance. Critically, HWF suppressed ecologically keystone taxa including *Glomus*, *Septoglomus*, and *Diversisporales* (LDA < −3.79), particularly in the topsoil, implying a functional shift away from mutualistic symbiosis.

### 3.4. AMF Communities Exhibit Heightened Sensitivity to Soil Nutrient Enrichment

**Figure 4:**
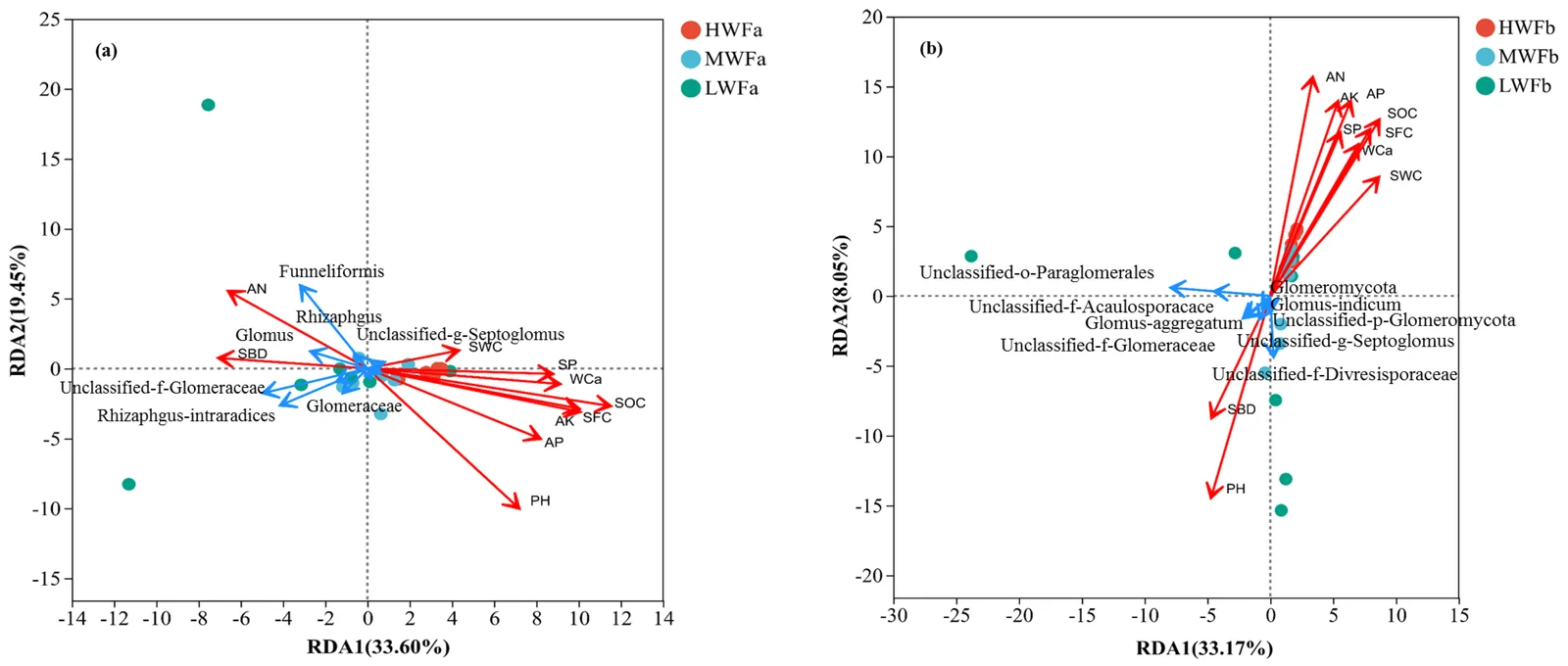
RDA of soil factors with AMF diversity, (**a**) for the topsoil and (**b**) for the subsoil. The supplementary Tables S4 and S5 report significance test of the Envfit for soil factors. The red arrows in the figure represent soil properties, and the blue arrows represent arbuscular mycorrhizal fungi genera. Soil parameters include pH, organic carbon (SOC), total nitrogen (TN), available phosphorus (AP), available potassium (AK), nitrate (NO_3_^−^-N), ammonium (NH_4_^+^-N), medium and micronutrient concentrations (Ca, Mg, Zn), as detailed in Section 2 (Materials and Methods).

We used Redundancy Analysis (RDA) to identify distinct edaphic drivers shaping AMF assembly in each soil layer (Figure 4a,b, Tables S3–S5). In the topsoil, soil organic carbon (SOC; r^2^ = 0.389, *p* = 0.004), available Zn (AZn), and available K (AK) were the primary predictors, with ruderal taxa (*Funneliformis*, *Rhizoglomus*) positively correlated with these labile resources. In contrast, subsoil AMF composition was predominantly governed by available N (AN; r^2^ = 0.426, *p* = 0.001), followed by SOC and available P (AP). Stress-tolerant lineages (*Glomus*, *unclassified* (*aff. Glomerales*, *Glomeromycetes*, *Glomeromycota*)) were strongly associated with these subsoil nutrient hotspots.

Notably, soil pH exerted opposing effects across depths: it negatively filtered acidophilic taxa (e.g., *Glomus aggregatum*) in the topsoil but positively correlated with subsoil community structure (r^2^ = 0.388, *p* = 0.009), likely reflecting intensified acidification from nitrification or cation leaching at depth.

### 3.5. Correlations Between AMF Genera Driven by Fertilization Ecosystem Mmultifunctionality in Peach Orchards

**Figure 5:**
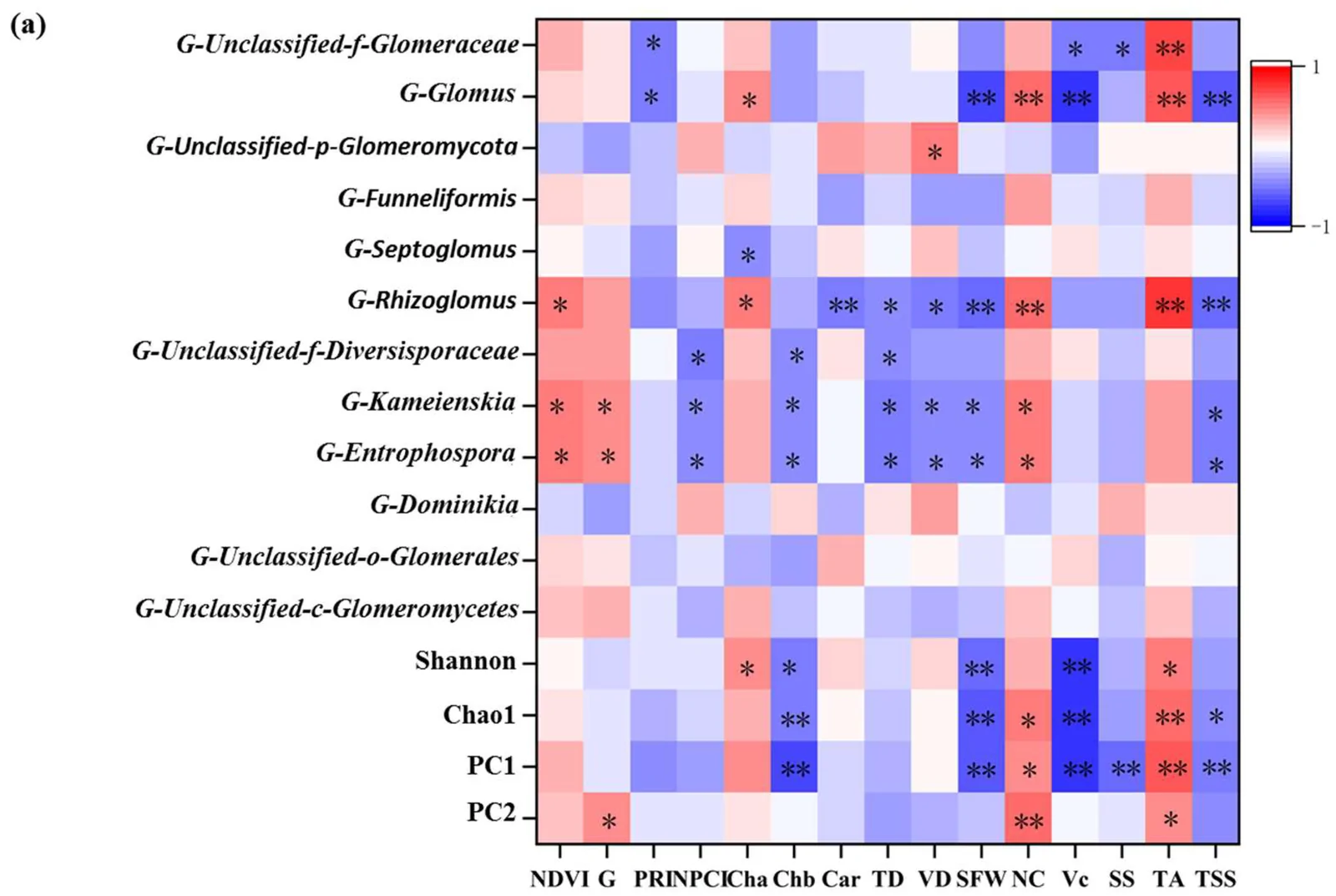

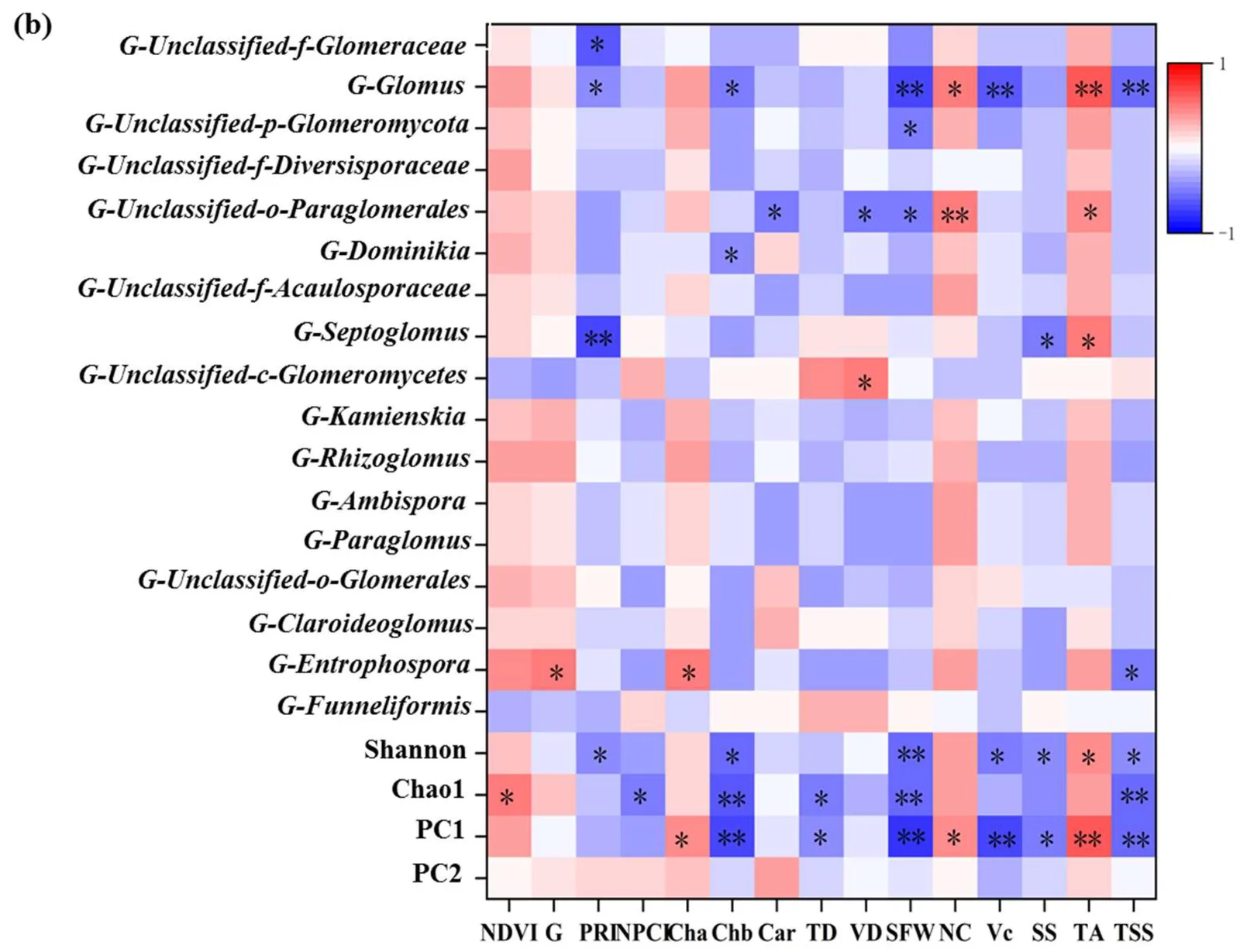
Correlations among AMF diversity/composition, dominant genera, foliar spectral indices, and peach yield/quality traits in the topsoil (**a**) and subsoil (**b**). Shannon and Chao1 represent the Shannon diversity index and Chao1 richness index of the AMF community, respectively. PC1 and PC2 denote the first two principal coordinates derived from the Principal Coordinates Analysis (PCoA) of the AMF community. Dominant AMF genera are defined as those with a relative abundance greater than 1% within the community. For detailed descriptions of the foliar spectral indices and peach yield/quality traits, please refer to Section 2.4. In all correlation matrices, blue and red colors indicate negative and positive correlations, respectively. Asterisks denote statistical significance levels (*, *p* < 0.05; **, *p* < 0.01).

Pearson correlation analysis indicated significant associations between AMF communities and both primary and economic productivity at different soil depths in the peach orchard (Figure 5a,b). In the topsoil (Figure 5a), the Shannon index was significantly positively correlated with Chb and Car content (*p* < 0.01, *p* < 0.05 respective). Conversely, both the Chao1 and PC1 exhibited extremely significant negative correlations with Chb, Car, VD, and TSS (*p* < 0.01). Additionally, the Chao1 index was extremely significantly negatively correlated with SFW (*p* < 0.01). The PC2 scores were only significantly positively correlated with G (*p* < 0.05). In the subsoil (Figure 5b), the correlation patterns of AMF diversity indices shifted markedly. The Shannon index was significantly negatively correlated with G, SFW, SS, TA, and TSS (*p* < 0.05), and positively correlated with VD and NC (*p* < 0.01). Similarly, the Chao1 showed primarily significant negative correlations with G, SS, TA, and TSS (*p* < 0.05 or *p* < 0.01). Chao1 and PC1 scores were also significantly positively correlated with VD and NC (*p* < 0.01) and negatively correlated with TSS (*p* < 0.01). This pattern suggests a potential trade-off wherein a more diverse or specific AMF community structure might influence carbon allocation patterns, favoring nitrogen assimilation at the expense of certain fruit quality metabolites.

Dominant AMF genera and leaf spectral parameters and fruit quality across soil depths. In the topsoil (Figure 5a), distinct dominant genera displayed differentiated correlation characteristics. *Kamienskia* and *Entrophospora* were significantly positively correlated with NDVI, G, Chb, Car, VD, NC, and TSS (*p* < 0.05, *p* < 0.01). Specifically, *Glomus* was significantly negatively correlated with VD, NC, and TSS (*p* < 0.01). Although *Rhizoglomus* was positively correlated with NDVI and Chb (*p* < 0.05, *p* < 0.01, respective), and negatively correlated with Car, VD, NC, and TSS (*p* < 0.01). Furthermore, *Septoglomus* was significantly positively correlated with Chb (*p* < 0.05). In the subsoil (Figure 5b), *Glomus* showed a significant positive correlation with G (*p* < 0.05) and extremely significant negative correlations with VD, NC, and TSS (*p* < 0.01), a trend consistent with that observed in surface soil. *Septoglomus* was significantly negatively correlated with G (*p* < 0.01), while showing significant positive correlations with VD and NC (*p* < 0.05, *p* < 0.01, respective). Notably, *Entrophospora* maintained a significant positive correlation solely with Chb (*p* < 0.05) in this layer, with no significant associations observed for other indicators.

### 3.6. Layer-Specific Effects of Fertilization, Soil Properties, and AMF Communities on Peach Productivity Based on PLS-SEM

**Figure 6:**
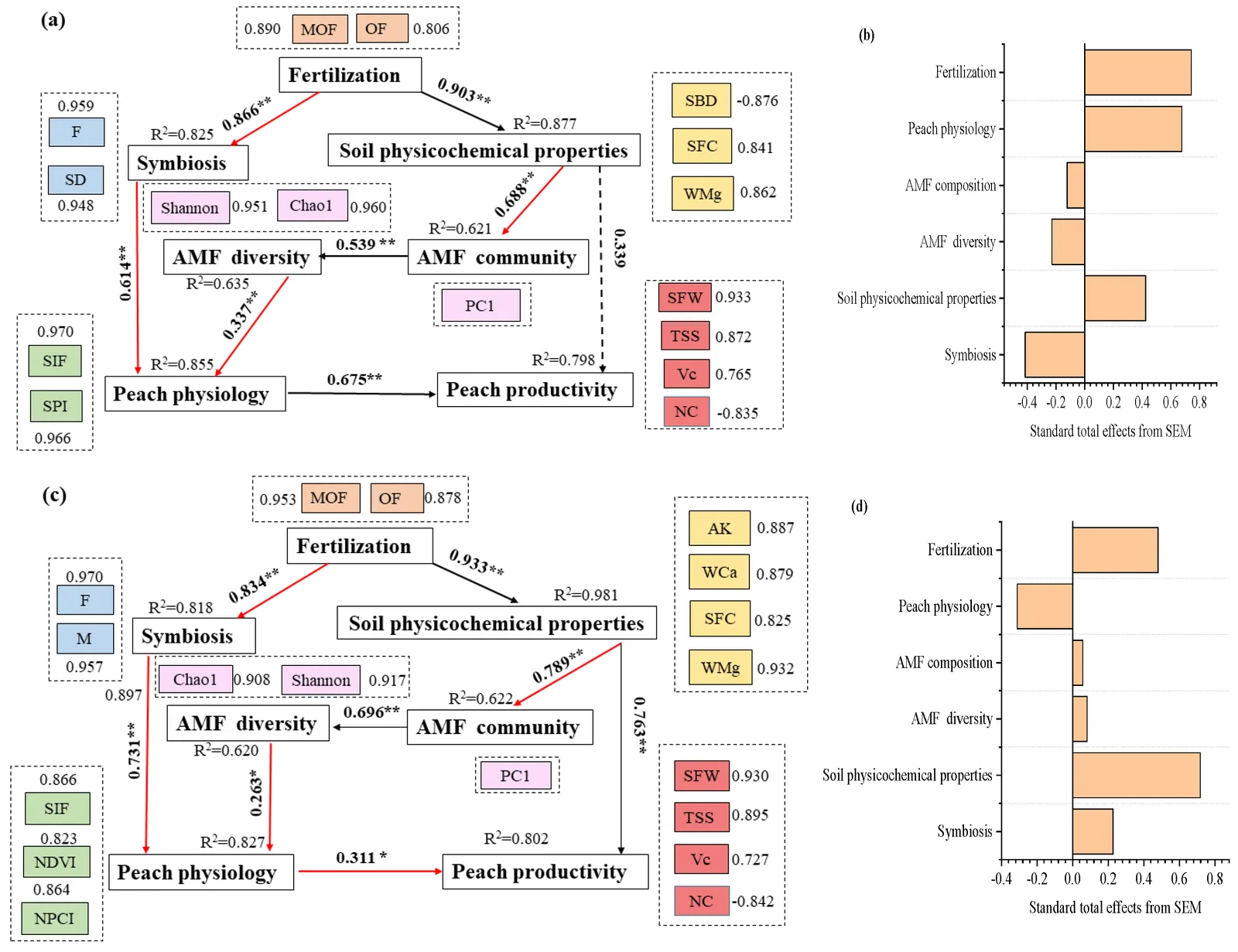
PLS-SEM for topsoil (**a**) and subsoil (**c**) layers, illustrating the interrelationships among OF and MF application, soil properties, AMF diversity and community composition, AMF symbiosis, peach tree physiology, and fruit yield. The coefficient of determination (R^2^) for each latent variable is indicated. Significant loadings (>0.7) of observed indicators on their respective latent variables are shown in adjacent boxes (e.g., OF = 0.953, SIF = 0.866). OF stands for organic fertilizer, and MOF stands for organic and Ca, Mg, Zn fertilizers. In addition, all loading factors have undergone multiple explicit checks, with VIF < 5 (Tables S6 and S7). Arrows represent hypothesized pathways among latent variables: solid black arrows indicate significant positive effects, solid red arrows denote significant negative effects, and dashed arrows represent non-significant relationships. Path coefficients are labeled alongside arrows, with * indicating *p* < 0.05 and ** indicating *p* < 0.01. Figures (**b**) and (**d**) display the total effects of each latent variable on yield in the topsoil and subsoil SEMs, respectively.

This study employed Partial Least Squares Structural Equation Modeling (PLS-SEM) to construct path models for topsoil (Figure 6a, Table S6) and subsoil (Figure 6c, Table S7) by integrating soil physicochemical properties, AMF community characteristics, and fruit yield data. Model validation confirmed the rationality of the latent variable structure. Path analysis revealed that fertilization significantly improved soil physicochemical properties (path coefficient β: 0.923–0.933) but strongly inhibited AMF symbiosis (β: −0.866 to −0.834). Soil physicochemical properties significantly reshaped AMF community composition and enhanced its diversity, whereas AMF diversity exhibited a significant negative impact on tree physiology during fruit maturation (β: −0.337 to −0.263).

Significant differences in productivity driving mechanisms were observed between soil layers: the direct driving effect of tree physiology on topsoil yield (β = 0.675) was much stronger than that on subsoil yield (β = 0.311) (Figure 6b). Conversely, subsoil physicochemical properties had a strong direct positive effect on yield (β = 0.763) (Figure 6d). Total effect analysis further revealed that topsoil productivity was primarily driven by the overall positive effect of fertilization, whereas subsoil productivity was dominated by soil physicochemical properties. Additionally, the AMF community and its diversity showed relatively positive contributions in the subsoil, highlighting the unique importance of subsoil AMF in orchard ecosystems.

## 4. Discussion

### 4.1. Impacts of Organic and Ca, Mg, Zn Fertilizers on AMF Symbiosis

This study found that the combined application of organic (MWF) significantly inhibited the root colonization rate (F %, M %), and spore density (SD) of AMF in peach orchard soils (Figure 2), which is partially consistent with our hypothesis. This aligns with meta-analytic evidence indicating that animal manures often suppress AMF more strongly than mineral fertilizers due to their high N and P content [7,25,44]. The underlying mechanism likely involves nutrient-mediated relaxation of plant dependence on mycorrhizal nutrient acquisition [14,45]. The synergistic input of mature commercial organic fertilizer and Ca, Mg, Zn fertilizers significantly increased the level of available soil nutrients, especially available zinc (AZn) and water-soluble magnesium (WMg) in the subsoil (Table S3) [3]. When soil phosphorus and nitrogen exceed plant demand, carbon allocation to AMF is downregulated, leading to reduced hyphal growth and sporulation [46]. While exogenous carbon sources may promote a more diverse AMF communities by supporting various niches [47], increased soil phosphorus levels reduce plant reliance on AMF for nutrient uptake [48,49], shifting from mutualism to parasitism or competition as carbon becomes a key determinant of symbiotic stability [46,50].

In our study, organic and Ca, Mg, Zn fertilizers in HWF further intensified this suppression and induced the most severe suppression of AMF colonization, reducing root colonization intensity by over 50% and spore density by 81.9% relative to LWF. Although the impact of Ca, Mg, Zn fertilizers on AMF remains understudied, our data suggest that direct fulfillment of host medium and micronutrient requirements may decrease the functional necessity of mycorrhizal uptake pathways [6]. Since sampling was conducted at a single time point in the fifth year after applying organic fertilizer and microelement fertilizer, which limits inference regarding temporal stability of AMF responses. This requires long-term fixed-location monitoring and evaluations to determine whether the suppression of organic and Ca, Mg, Zn fertilizers on AMF symbiosis is time-dependent.

### 4.2. Organic and Ca, Mg, Zn Fertilizers Were Mediators of AMF Functional Genera

Our results show that HWF and MWF significantly reduce the abundance of *Rhizoglomus* and *Kamienskia*, and significantly increase the abundance of *Glomus* and *Dominkia* (*p* < 0.05) (Figure 3, Figure 4 and Figure S4). These changes can be interpreted through the lens of AMF life-history strategies. *Glomus* are typically classified as competitive (C-) or ruderal (R-) strategists, investing heavily in extensive extraradical hyphae for efficient P and N scavenging [51,52,53]. High nutrient may lead to carbon reallocation away from AMF and subsequent decline of these taxa. In contrast, the relative persistence of certain *Diversisporales* and *unclassified_Dominikia* in the subsoil under MWF and HWF may reflect stress-tolerant (S-) traits, such as lower metabolic demands or adaptation to chemically heterogeneous microsites in subsoil [16]. These imply a community-wide shift toward low-investment symbioses under fertilization, consistent with the “mutualism–parasitism continuum” framework [12,54].

LEfSe analyses further confirmed depth-specific indicator taxa. The relative abundance of ruderal genera such as *Funneliformis* and *Rhizoglomus* was higher in the nutrient-rich topsoil, whereas stress-tolerant lineages (*Glomus*, *unclassified* (*aff. Glomerales*, *Glomeromycetes*, *Glomeromycota*)) dominated subsoil nutrient hotspots. *Rhizoglomus*, which is a key genus for phosphorus mobilization [54,55], pronouncedly declined in the subsoil under HWF, suggesting potential functional degradation of the mycorrhizal network under higher fertilization, particularly in the subsoil where plants rely heavily on AMF for nutrient access. Certain species of *Glomus* that are associated with zinc uptake and redistribution within the plant [6,56,57] also decreased. These results imply a shift towards more stress-tolerant and possibly less dependent associations with soil microbial communities under altered nutrient conditions. With the growing adoption of ASV approaches as an emerging standard, the implementation of high-resolution methods in future investigations may resolve the fine-scale AMF diversity potentially obscured by conventional 97% similarity clustering thresholds.

### 4.3. Depth-Dependent Environmental Filtering Shapes AMF Community

The AMF community in the peach orchard showed significant vertical stratification characteristics (Figure 3). Compare to the topsoil, the subsoil AMF community was more sensitive to fertilization treatments In our research, the subsoil has a stronger buffering capacity, and the nutrient accumulation and pH changes caused by fertilization have a more significant screening effect on AMF. At the same time, the exudate input from the deep roots of peach trees may also reshape the ecological niche of subsoil AMF [29]. Indeed, agricultural practices leave an indelible imprint on AMF communities through both direct and indirect edaphic modifications [10,12,16]. Trenching and back filling during fertilization increased SFC, which correlated positively with AMF diversity—a finding consistent with the role of AMF hyphae in stabilizing soil aggregates [1,7,12,14] and the known prevalence of *Glomeraceae* in clay-rich matrices [58]. Yet perhaps more consequential is the nutrient-mediated suppression of symbiosis. Our orchard soils exhibited AP concentrations far exceeding the 8–15 mg/kg threshold optimal for mycorrhizal P uptake [44,45,46,49], providing a mechanistic explanation for reduced colonization and sporulation. Crucially, pH exerted opposing effects across depths: it negatively correlated with acidophilic taxa (e.g., *Glomus aggregatum*) in the topsoil but positively structured subsoil communities. This reversal likely stems from intensified nitrification and cation leaching at depth, leading to stronger selective pressure in less buffered subsoil environments. This depth-related sensitivity suggests that subsoil AMF communities, while studied less often, might be more vulnerable to human-induced disturbances. The heightened adverse reactions in the subsoil may be linked to decreased intrinsic nutrient diffusion rates, diminished microbial competition, or unique physiological adaptations that make these communities more susceptible to changes in nutrient availability. The ongoing reduction of key genera like *Rhizoglomus*—recognized for its significant functional responsiveness and efficiency in phosphorus mobilization—strongly suggests a possible functional decline of the mycorrhizal network under heavy fertilization practices.

While our study revealed pronounced vertical stratification in a mature peach orchard, similar patterns have emerged across diverse ecosystems—yet with system-specific nuances [11,22,25,47,51]. This cross-system perspective implies that our findings may be generalizable to perennial cropping systems, rather than to annual or heavily managed agroecosystems.

### 4.4. Trade-Offs Between AMF Communities and Ecosystem Multifunctionality in Peach Orchards

Correlation analysis demonstrated close associations between AMF community metrics (diversity, PC1 and PC2) and peach physiological spectral indices, as well as fruit yield and quality traits, indicating that AMF communities are closely linked to orchard ecosystem functioning [8,14]. Stronger correlations between subsoil AMF taxa and ecosystem functional variables suggest that subsoil microbial characteristics are potentially more closely coupled to orchard performance than surface soil communities. These observations imply that subsoil horizons constitute a key zone for plant–soil feedbacks in peach orchards, where AMF community characteristics are more sensitive to fertilization management and tightly associated with tree growth and fruiting performance. Consistent with previous studies, optimized management of soil AMF communities is likely conducive to improving orchard ecosystem functions [8,14,44], while the present findings further reveal the complexity of plant–AMF associative relationships under field fertilization conditions. The high sensitivity of subsoil AMF communities supports the view that the subsoil acts as a potential functional pool, highlighting its indispensable ecological value in sustaining orchard ecosystem stability.

PLS-SEM results indicated that organic and Ca, Mg, Zn fertilization levels were statistically associated with reduced AMF symbiosis intensity, which was presumably linked to soil nutrient enrichment. Variations in AMF community composition (primarily tolerant taxa) were further correlated with plant C/N resource allocation patterns and mild variations in leaf physiological activity (SIF/NDVI), which were synchronously associated with improved fruit productivity. Such correlative tendencies were more prominent in the subsoil layer, where favorable soil conditions (high SWC, AK, and WMg) were concurrently associated with superior yield performance and stable AMF microhabitats, potentially enabling AMF-related resource allocation patterns even under relatively low symbiosis levels. The present results imply a potential trade-off in plant–AMF associations: differentiated AMF community structures may correlate with altered plant carbon allocation strategies, and nutrient-induced shifts in symbiotic status may associate with transitions along the mutualism–parasitism/competition continuum [3,15,46]. This study attempts to achieve cross-scale integration from the soil environment to plant phenotypes and to economic output. We not only measured physicochemical factors of different soil layers (such as pH, SOC, AZn, WMg), AMF community composition, and functional indicators (root colonization rate, spore density), but also simultaneously obtained hyperspectral vegetation indices reflecting the physiological status of peach trees (such as NDVI, PRI; see Table S2), and associated them with final fruit yield attributes. As a non-destructive proxy for plant physiological status, spectral indices can successfully bridge underground microbial processes with aboveground productivity [59], highlighting the potential of multi-source data fusion in smart orchard management. Although this study constructed a multidimensional correlation framework and revealed key pathways, certain limitations remain: First, the study was based on a 5-year field experiment, which is a short-term effect and cannot reflect the long-term evolution pattern and functional stability of the AMF community. Second, the flux of AMF-mediated carbon-nitrogen-phosphorus cycles was not quantified, and the physiological mechanism of ‘how AMF regulates plant resource allocation’ was not deeply analyzed. Third, the research area was limited to the peach orchard in Yanqing, Beijing, and the generalizability of the results needs to be verified in different climate zones and different orchard types. Future work should integrate root phenology, isotopic tracing, and targeted inoculation experiments to causally test the contributions of depth-stratified AMF taxa to carbon allocation, nutrient acquisition, and agroecosystem resilience.

## 5. Conclusions

This study integrated stratified soil physicochemical assessment, AMF community analysis, hyperspectral physiological monitoring, and multiple statistical approaches (correlation analysis, RDA, and PLS-SEM) to explore the associations between organic–and Ca, Mg, Zn fertilizers, AMF symbiotic and community traits and function of arbuscular mycorrhizal fungi (AMF) in perennial fruit orchards. Notably, all findings highlight distinct depth-dependent plant–AMF associations in perennial orchard systems. Fertilization-induced soil nutrient enrichment was strongly associated with suppressed AMF root colonization and spore density, and significant shifts in AMF community composition. Higher fertilization correlated with the decrease of nutrient-sensitive *Rhizoglomus* and the enrichment of stress-tolerant *Glomus*, suggesting a potential transition across the plant–AMF mutualism–parasitism continuum. RDA and correlation analyses verified significant vertical stratification of AMF communities. Subsoil AMF assemblages were more responsive to fertilization disturbance and exhibited tighter correlations with orchard functional performance, indicating that subsoil serves as a crucial functional reservoir for sustaining orchard microbial stability. PLS-SEM further constructed a robust correlative framework how the shifts of AMF driven by fertilization affect orchard multifunction. At the fruit maturation stage, fertilization-related reduction in AMF symbiosis was statistically associated with modified plant C/N allocation strategies. Community-level AMF variations were correlated with attenuated leaf physiological activity but enhanced fruit yield, implying a clear trade-off between tree vegetative performance and reproductive productivity, with this pattern being more prominent in the subsoil. In our study, the single-stage sampling and 5-year field trial cannot capture the long-term temporal dynamics of AMF communities. Furthermore, the observed correlative associations lack quantitative validation of nutrient cycling processes and in-depth mechanistic interpretation, and the results are restricted by site-specific conditions. Future research combining isotopic tracing and microbial inoculation assays will help clarify the potential mechanisms by which depth-stratified AMF communities modulate plant resource allocation in orchard ecosystems. This study only collected soil and plant samples once at the fruit ripening stage, and the five-year fixed field trial cannot reflect the long-term temporal dynamics of AMF communities under fertilization. Although PLS-SEM were constructed to reveal correlative pathways, the causal regulatory mechanisms between depth-stratified AMF communities and tree carbon-nitrogen allocation remain unvalidated via isotope tracing and inoculation tests; furthermore, the experiment was limited to peach orchards in Yanqing, Beijing, and the universality of the results requires verification across different climate zones and orchard types.

## Figures and Tables

**Table 1:** Field Regimes of Organic and Medium and Micronutrient Fertilizers for Peach Trees.

Regime	Chemical Fertilizer (N:P_2_O_5_:K_2_O = 15:5:10)	Organic Fertilize (47.6% SOC N + P + K = 5%)	Ca, Mg, and Zn Fertilizers		
			Ca (OH)_2_	ZnSO_4_	MgSO_4_
LHW	900 kg·ha^−1^	0	0	0	0
MWF	900 kg·ha^−1^	3 t·ha^−1^	0	0	0
HWF	900 kg·ha^−1^	3 t·ha^−1^	20 kg·ha^−1^	4 kg·ha^−1^	15 kg·ha^−1^

Note: Organic fertilizer was fully decomposed from pig manure and maize straw (Beijing Beilang Zhong Organic Fertilizer Factory, Beijing, China), with a 47.6% organic matter content and a total nutrient content (N, P_2_O_5_ and K_2_O) of 5%. The amounts of chemical fertilizers, organic fertilizers, and Ca, Mg, Zn fertilizers are determined based on the conventional fertilization rates in local peach orchards.

**Table 2:** Effects of Fertilization on Foliar Spectral Traits in the Peach Orchard.

Regime	NDVI	GI	OSAVI	PRI	NPCI	MCARI	SIPI
LWF	0.46 ± 0.060 b	1.77 ± 0.116 c	0.51 ± 0.367 b	0.030 ± 0.0057 b	0.027 ± 0.010 b	0.24 ± 0.038 a	0.58 ± 0.042 b
MWF	0.53 ± 0.047 a	1.84 ± 0.120 b	0.52 ± 0.046 b	0.035 ± 0.0070 b	0.028 ± 0.015 b	0.21 ± 0.041 b	0.56 ± 0.052 b
HWF	0.57 ± 0.043 a	1.93 ± 0.140 a	0.58 ± 0.041 a	0.042 ± 0.0069 a	0.043 ± 0.010 a	0.21 ± 0.052 b	0.63 ± 0.043 a

Note: Values are means ± SD of seven replicates. Different lowercase letters within each column indicate significant differences among treatments at *p* < 0.05 (one-way ANOVA with Tukey’s HSD post hoc test). The coefficient of variation (CV) values of NDVI, GI, OSAVI, PRI, NPCI, Cha, Chb, and SIPI were 9.76%, 7.26%, 7.78%, 14.18%, 41.13%, 3.90%, 10.31%, and 8.09%, respectively.

**Table 3:** Effects of Fertilization on Peach Fruit Size, Weight and Quality.

Regime	TD (mm)	VD (mm)	SFW (g)	NC (mg kg^−1^)	Vc (mg kg^−1^)	SS (%)	TA (%)	TSS (%)
LWF	70.10 ± 0.18 b	65.79 ± 0.51 b	168.50 ± 4.72 b	119.79 ± 5.43 a	58.84 ± 4.73 b	8.48 ± 0.27 b	2.35 ± 0.21 a	9.64 ± 0.12 b
MWF	73.56 ± 0.30 a	71.10 ± 0.38 a	179.12 ± 6.18 b	103.14 ± 8.86 b	64.53 ± 9.46 b	9.50 ± 1.09 a	2.01 ± 0.24 a	10.55 ± 0.18 a
HWF	73.99 ± 0.57 a	73.33 ± 0.82 a	196.04 ± 5.80 a	90.40 ± 2.57 c	93.00 ± 5.51 a	10.2 ± 0.596 a	1.51 ± 0.11 b	10.58 ± 0.085 a

Note: Values are means ± SD of seven replicates. Different lowercase letters within each column indicate significant differences among treatments at *p* < 0.05 (one-way ANOVA with Tukey’s HSD post hoc test). The coefficient of variation (CV) values of TD, VD, SFW, NC, VC, SS, TA and TSS were 4.80%, 5.60%, 11.01%, 22.63%, 10.47%, 14.76%, and 4.23%, respectively.

## Data Availability

The obtained gene sequences from Illumina platform are now deposited in the National Center for Biotechnology Information (NCBI) Sequence Read Archive with an accession number PRJNA835607. The data presented in this study are available on request from the corresponding author.

## Supplementary Materials

The following supporting information can be downloaded at: https://www.mdpi.com/article/10.3390/jof12080543/s1, Figure S1: The diagram of trenching beside peach trees (a) and the layout of regime treatments in the peach orchard (b). Figure S2: Venn diagrams illustrating the shared and unique AM fungal OTUs between topsoil and subsoil. Figure S3: AMF alpha-diversity indices (OTU numbers, Chao1, Shannon, Simpson) in (a) topsoil and (b) subsoil. Figure S4: Significance test of AMF abundance at the genus level in topsoil (a) and subsoil (b) under different treatments. Figure S5: Results of the Linear discriminant analysis Effect Size (LEfSe) identifying AMF taxa differentially abundant under specific treatments and soil depths. Table S1: Pre-treatment soil properties in the experimental peach orchard. Table S2: Equations and references of foliar spectral indexes. Table S3: Soil physicochemical properties in the experimental peach orchard of different regimes. Table S4: Envifit value of soil factors in RDA analysis of AMF genus in the Topsoil. Table S5: Envifit value of soil factors in RDA analysis of AMF genus in the Subsoil. Table S6: VIF Values of Each Load Factor in SEM of the Topsoil. Table S7: VIF Values of Each Load Factor in SEM of the Subsoil.
