# Supplementary material for: Co-Application of Organic and Ca, Mg, Zn Fertilizers Reshapes Depth-Stratified Arbuscular Mycorrhizal Fungal Communities in Orchard Soil
Source: J Fungi (Basel). 2026 Jul 23;12(8):543. doi: 10.3390/jof12080543 (PMC13514299; doi:10.3390/jof12080543)
Supplement: Supplementary file 1 [file jof-12-00543-s001.zip › jof-4323397-supplementary.pdf]

Supplementary material

Supplementary figures

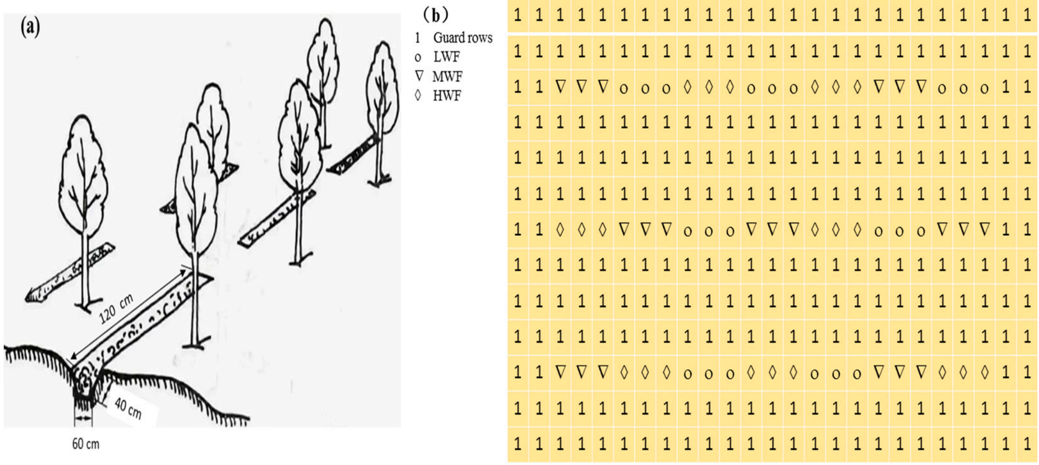

Figure S1 The diagram of trenching beside peach trees (a) and the layout of regime treatments in the peach orchard (b).

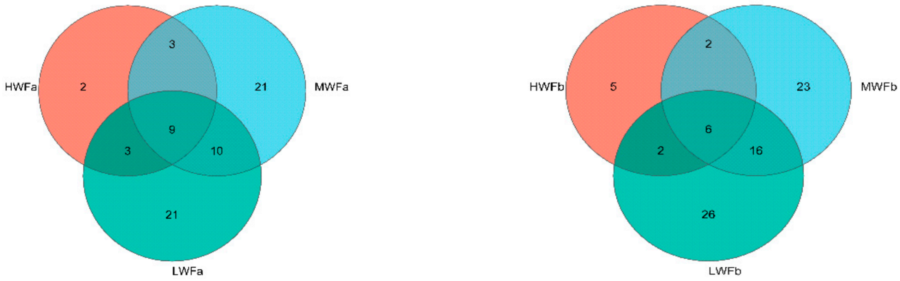

Figure S2 Venn diagrams illustrating the shared and unique AM fungal OTUs between the topsoil and the subsoil.

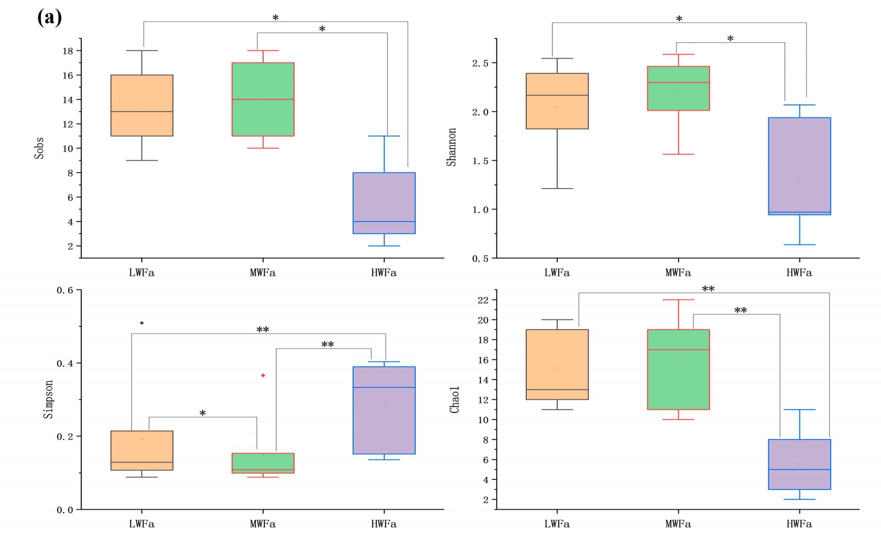

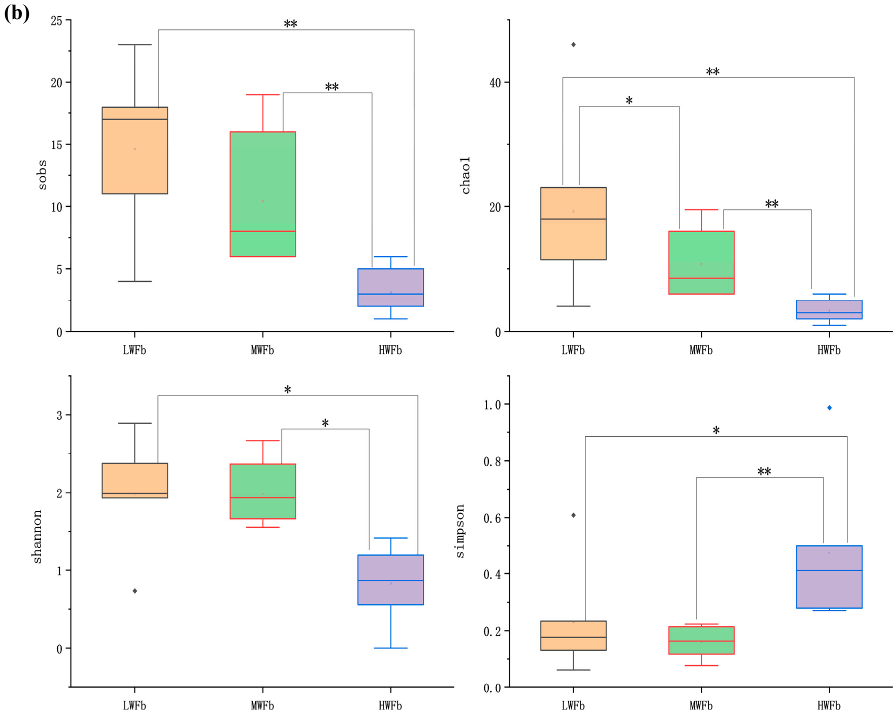

Figure S3 AMF alpha-diversity indices (OTU numbers, Chao1, Shannon, Simpson) in (a) topsoil and (b) subsoil. Asterisks indicate significant differences between the HWF treatment and the LWF control within each depth (*, $P < 0.05$; **, $P < 0.01$; Student's t-test).

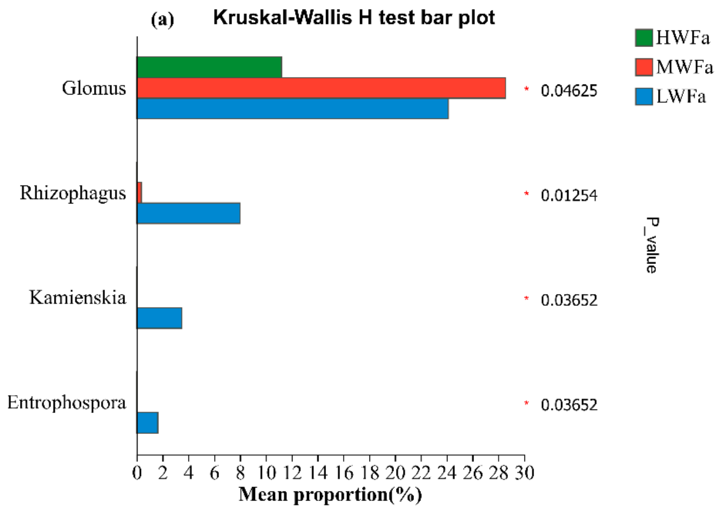

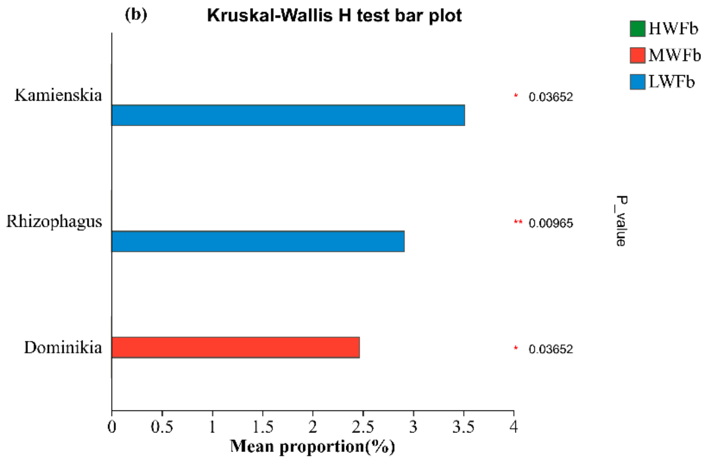

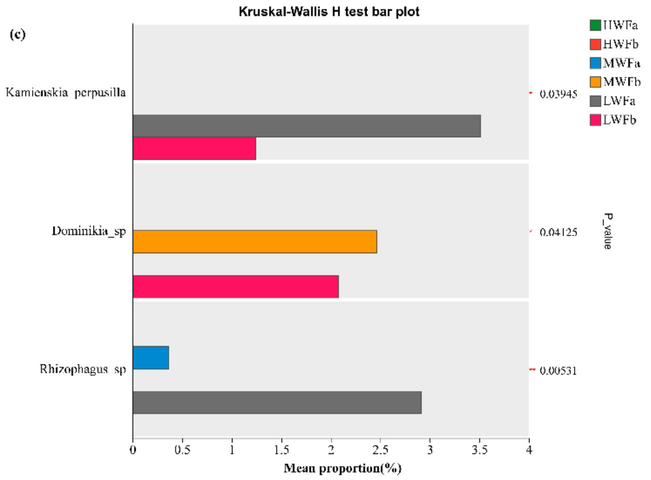

Figure S4 Significance test of AMF abundance at the genus level in topsoil (a) and subsoil (b) under different treatments. Kruskal-Wallis rank test was used, and post-hoc test with Tukey-Kramer method. *, P < 0.05; **, P < 0.01.

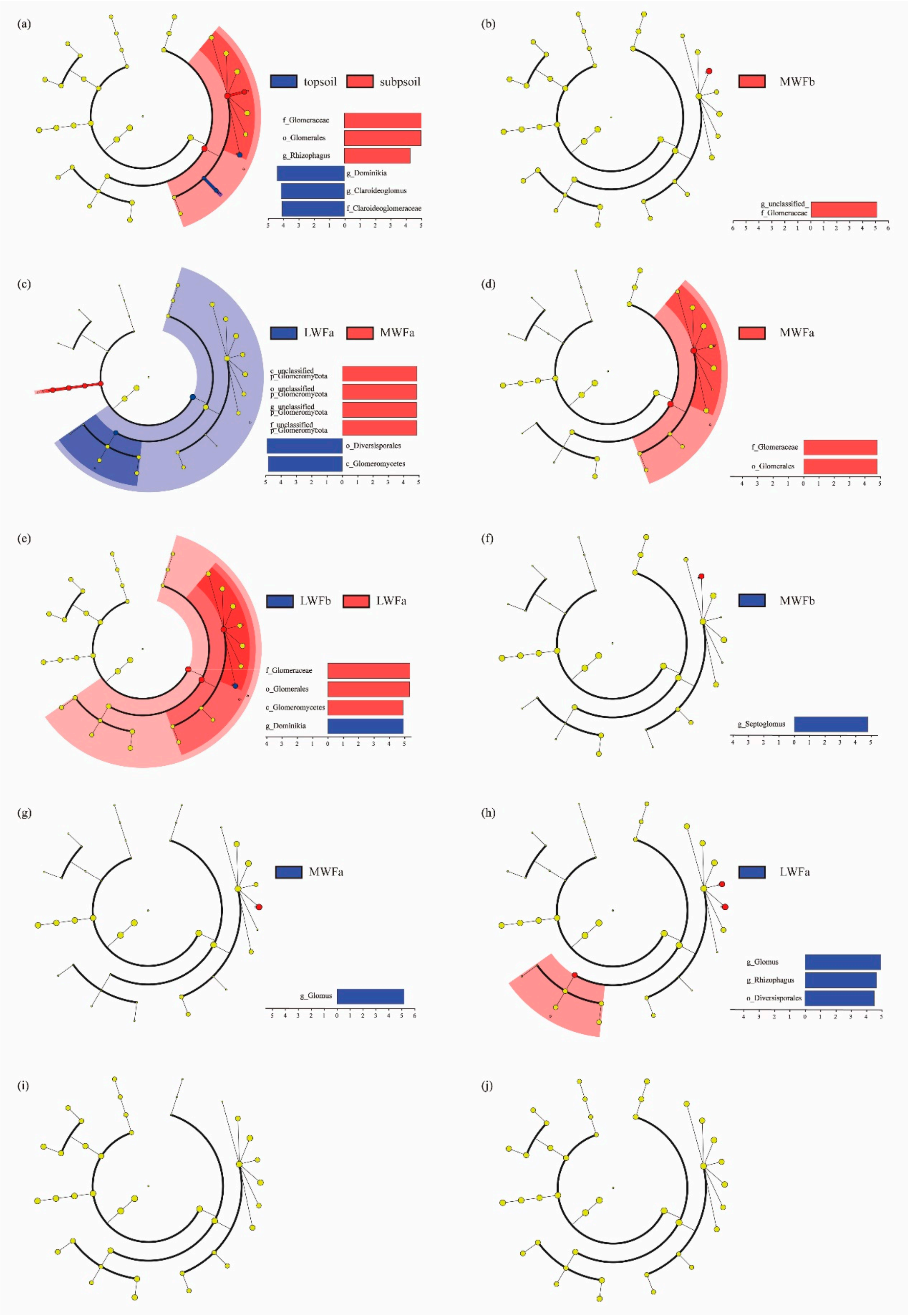

Figure S5 Results of the Linear discriminant analysis Effect Size (LEfSe) identifying AMF taxa differentially abundant under specific treatments and soil depths. a represents the difference between topsoil and subsoil; b represents the difference between HWFb and MWFb; c represents the difference between LWFa and MWFa; e represents the difference between LWFa and LWFb; f represents the difference between HWFb and MWFb; g represents the difference between HWFa and MWFa; h represents the difference between HWFa and LWFa; i represents the difference between HWFb and LWFb; j represents the difference between HWFa and HWFb.

Supplementary Tables

Table S1. Pre-treatment soil properties in the experimental peach orchard

Soil Depth	AN $mgkg^{-1}$	AP $mgkg^{-1}$	AK $mgkg^{-1}$	TN %	TP %	TK %	SOC gkg^{-1}	CEC $cmolkg^{-1}$	WCa $cmolkg^{-1}$	WMg $cmolkg^{-1}$	AZn mmolkg-1
0-20cm	105	81.9	457.67	0.17	0.10	2.03	13.23	9.42	0.31	0.12	0.60
20-40cm	90.47	19.77	133.33	0.11	0.06	1.98	10.96	9.35	0.24	0.06	0.15

Note, AN: lkali-hydrolysable N; AP: available P; AK: available K; TN: total N; TP: total P; Tk: total K, SOC: soil organic carbon; WCa: water soluble Ca; WMg: water soluble Mg; AZn: available Zn. The same below.

Table S2. Equations and references of foliar spectral indexes

Spectral Index	Equation	Reference
NDVI	$(R_{800}-R_{680})/(R_{800}+R_{680})$	Rouse et al. (1974)
GI	R_{554}/R_{667}	Gitelson et al. (1996)
OSAVI	$(1+0.16)*(R_{790}-R_{670})/(R_{790}-R_{670}+0.16)$	Rondeaux et al. (1996)
PRI	$(R_{531}-R_{570})/(R_{531}+R_{570})$	Gamon et al. (1992)
NPCI	$(R_{680}-R_{430})/(R_{680}+R_{430})$	Peñuelas et al. (1994)
MCARI	$[(R_{700}-R_{670})-0.2*(R_{790}-R_{550})]*(R_{700}/R_{670})$	Daughtry et al. (2000)
SIPI	$(R_{790}-R_{450})/(R_{790}+R_{650})$	Peñuelas et al. (1995)

Note： R800, R680, R554, R667, R790, R670, R531, R570, R430, R550, R450, and R650 represent the spectral reflectance at wavelengths of 800, 680, 554, 667, 790, 670, 531, 570, 430, 550, 450, and 650 nanometers (nm), respectively.

Table S3. Soil physicochemical properties in the experimental peach orchard of different regimes

Depth （cm ）	Regimes	SBD (gcm^{-3})	SP (%)	SOC (gkg^{-1})	SFC (%)	SWC (%)	pH	AN ($mgkg^{-1}$)	AP (mg kg^{-1})	AK ($mgkg^{-1}$)	WCa ($cmolkg^{-1}$)	WMg ($cmolkg^{-1}$)	AZn ($mmolkg^{-1}$)
	LWFa	1.47a	44.64b	18.02b	23.24b	18.40b	7.45a	81.69a	52.41a	138.05c	0.27b	0.10b	8.40c
0 ~ 20	MWFa	1.41a	44.70b	19.18ab	29.71a	19.77ab	7.61a	81.43a	33.94b	269.78b	0.27b	0.10b	10.30b
	HWFa	1.35b	48.89a	21.25a	30.46a	21.40a	7.5a	69.35b	41.39ab	378.04a	0.39a	0.16a	21.40a
	LWFb	1.57a	41.01b	16.21c	23.81b	18.94b	7.91a	73.33a	23.97b	115.02c	0.18b	0.04b	6.31b
20 ~ 40	MWFb	1.56a	40.87b	17.83b	28.24a	18.72b	7.81a	61.90b	27.58a	155.91b	0.18b	0.06a	8.60ab
	HWFb	1.44b	45.80a	18.37a	28.60a	20.26a	7.48b	53.04c	29.38a	258.21a	0.27a	0.07a	16.80a

Note: Values are means of seven replicates. Different lowercase letters within each column indicate significant differences among treatments at $p< 0.05$ (n=7; one-way ANOVA with Tukey’s HSD post hoc test)

Table S4. Envifit value of soil factors in RDA analysis of AMF genus in the Topsoil

	RDA1	RDA2	r2	P_value

SOC	0.99945	-0.03317	0.38869	0.004
AZn	0.99999	0.00428	0.34908	0.007
AK	0.99771	-0.0677	0.29444	0.011
SFC	0.99669	-0.08125	0.29821	0.028
WCa	0.99929	0.03758	0.24118	0.047

Table S5. Envifit value of soil factors in RDA analysis of AMF genus in the Subsoil

	RDA1	RDA2	r2	P_value
AN	0.27768	0.96067	0.42636	0.001
SOC	0.61194	0.79091	0.40848	0.002
SFC	0.60084	0.79937	0.35685	0.006
AP	0.47655	0.87915	0.40219	0.006
WMg	0.62295	0.78226	0.4251	0.006
AK	0.42408	0.90563	0.37783	0.007
AZn	0.52514	0.85102	0.39623	0.009
PH	-0.37551	-0.92682	0.38803	0.009
WCa	0.59091	0.80673	0.28727	0.024
SP	0.48772	0.873	0.28566	0.029
SWC	0.73897	0.67374	0.2578	0.04

Table S6. VIF Values of Each Load Factor in SEM of the Topsoil

Factor	VIF
OF	1.250
MF	1.250
Shannon	3.132
Chao1	3.132
PC1	1
F	3.021
SD	3.021
SBD	2.238
SFC	1.800
WMg	1.794
SPI	4.249
SIF	4.249
NC	2.342
SFW	4.674
Vc	2.445
TSS	3.611

Table S7. VIF Values of Each Load Factor in SEM of the Subsoil

Factor	VIF
OF	1.333
MF	1.333
Shannon	1.795
Chao1	1.795
PC1	1
F	3.793
M	3.793
AK	3.151
SFC	2.192
WCa	2.873
WMg	4.003
NDVI	1.956
SIF	1.578
NPCI	2.279
NC	2.342
SFW	4.674
Vc	2.445
TSS	3.611

References:

Rouse, J. W.; Haas, R. H.; Schell J A, Deering D W. *Monitoring the vernal advancement and retrogradation (green wave effect) of natural vegetation*. In: NASA/GSFC, Type III Final Report, Greenbelt, MD, USA, 1974. pp 371–373.

Gitelson, A.A.; Merzlyak, M.N. 1996. Use of a green channel in remote sensing of global vegetation from EOS-MODIS. *Remote Sens. Environ.* 1996; 58(3):289-298. http: //doi.org/10.1016/S0034-4257(96)00072-7.

Rondeaux G.; Baret F.;Steven M.1996.Optimization of Soil-Adjusted Vegetation Indices. *Remote Sens. Environ.*55, *2*, 95-107. http: //doi.org/10.1016/0034-4257(95)00186-7.

Gamon, J.A., Field, C.B., Bilger, W., Bjorkman, O., Fredeen, A.L., Oenuelas, J. 1990. Remote sensing of the xanthophyll cycle and chlorophyll fluorescence in sunflower leaves and canopies. *Oecologia*, 85,1–7. https://doi.org/10.1007/BF00317336.

Peñuelas J.; Gamon J.A.; Fredeen A.L.; Merino J.; Field C.B.1994. Reflectance indices associated with physiological changes in nitrogen- and water-limited sunflower leaves. *Remote Sens. Environ.*48, *2*,135-146. http://doi.org/10.1016/0034-4257(94)90136-8.

Daughtry, C.S.T; Walthall, C.L; Kim, M.S; Colstoun, E.Brown de; McMurtrey, J.E.2000.Estimating Corn Leaf Chlorophyll Concentration from Leaf and Canopy Reflectance. *Remote Sens.Environ.*74,*2*,229-239.http://doi.org/10.1016/S0034-4257(00)00113-9.

Peñuelas, J.; Filella, I.; Gamon, J.A. 1995. Assessment of photosynthetic radiation-use efficiency with spectral reflectance. *New Phytol.* 131,291–296. https://doi.org/10.1111/j.1469-8137.1995.tb03064.x.
